# Anxiety, Stress, and Social Support in Pregnant Women in the Province of Leon during COVID-19 Disease

**DOI:** 10.3390/healthcare10050791

**Published:** 2022-04-24

**Authors:** Rubén García-Fernández, Cristina Liébana-Presa, Pilar Marqués-Sánchez, María Cristina Martínez-Fernández, Natalia Calvo-Ayuso, Pedro Hidalgo-Lopezosa

**Affiliations:** 1 SALBIS Research Group, Faculty of Health Sciences, Campus de Ponferrada, Universidad de León, 24401 León, Spain; rgarcf@unileon.es (R.G.-F.); pilar.marques@unileon.es (P.M.-S.); mmartf@unileon.es (M.C.M.-F.); 2 ESVITER Research Group, Faculty of Health Sciences, Campus de Ponferrada, Universidad de León, 24401 León, Spain; ncala@unileon.es; 3 Faculty of Medicine and Nursing, Universidad de Córdoba, 14004 Córdoba, Spain; phlopezosa@uco.es

**Keywords:** anxiety, COVID-19, pregnancy, social support, stress

## Abstract

The COVID-19 pandemic has posed a high risk to the mental health of the entire population. Pregnant women are strongly affected by the consequences of COVID-19, resulting in increased anxiety and stress. Social support can be a protective factor when it comes to mental health disturbances such as anxiety, fear, or stress in pregnant women. This research aims to describe the anxiety and stress of women in the first trimester of pregnancy in times of pandemic and its relationship with social support. A quantitative, descriptive, cross-sectional study was conducted. A total of 115 women in the first trimester of pregnancy participated. Anxiety was found in 78.3% of the women. Self-perceived stress correlated significantly with the dimensions “concern for changes in oneself”, “feelings about oneself”, “concern about the future”, and very weakly with social support. In addition, a negative correlation was observed between “feelings about oneself” and social support. During the COVID-19 pandemic, anxiety levels of women in our population are elevated. Pregnant women during the first trimester of pregnancy showed higher levels of fear of childbirth and concern about the future than multiparous women. Increased social support and decreased stress seem to influence “feeling about oneself”.

## 1. Introduction

In December 2019, SARS-CoV-2 was described for the first time in Wuhan, Hubei Province, China [1]. Since then, coronavirus infection (COVID-19) has spread rapidly around the world, prompting the World Health Organization to declare a global pandemic on 11 March 2020 [1]. This disease causes a pneumonia that has spread rapidly around the world [2].

SARS-CoV-2 disease has caused an increase in anxious symptoms in the population, mainly due to fear of contagion [3,4,5]. As a consequence, the impact of physical and social isolation, coupled with the COVID-19 pandemic, has posed a high risk to the mental health of the population. During epidemics and pandemics, factors such as fear of death and alterations in daily activities due to the need for quarantine can have significant psychological consequences, leading to depression and anxiety [6,7]. Specifically, during COVID-19, the risk to psychological health is severe, with anti-anxiety and depression affecting approximately one-third of the population [8]. The COVID-19 pandemic is a collective trauma that threatens the mental health of citizens, leading to increased emotional stress, reduced social support, and increased risk of affective symptoms [8,9].

COVID-19 affects pregnant women to a greater extent because they are more vulnerable to pulmonary infections due to physical changes in the respiratory system and maternal immunosuppression [10,11,12]. Pregnant women are therefore considered a high-risk group, which adds to the general population’s apprehension and concern about developing COVID-19 [10,12].

Women are not only afraid of becoming infected with COVID-19, but they are also very concerned about the well-being of the fetus. Health authorities are asking the population to stay away from health care facilities [7]. However, women will have to go to the hospital to have their baby sooner or later [7]. The prevalence of stress and anxiety in pregnancy is known to be increased compared to the general population [13,14]. Pregnant women may have more anxious symptoms than the general population, either because of the time they are pregnant or because they are worried that something bad might happen to the fetus [15]. Different studies have reported the relationship of pregnancy-related anxiety and health-related problems such as intrauterine growth retardation, premature birth, cleft lip and palate, neonatal death, autism, hyperactivity, and other neurodevelopmental disorders [6,8].

The literature on fear of childbirth reveals that it is observed in all pregnant women, whether nulliparous, primiparous, or multiparous, and can have consequences for their health as well as for the labor and puerperal period [7]. Fear of childbirth is combined with childbirth-related anxiety and in turn is specifically related to the COVID-19 pandemic [7]. It should be noted that fear of childbirth is used as a general label for many types of anxieties related to women’s experience of pregnancy, childbirth, and their outcomes [16].

Women with higher perceived stress are more likely to be anxious than those with lower perceived stress [12,17,18]. In addition, there is evidence that pregnant women have experienced high levels of anxiety and depressive symptoms during the pandemic [19,20,21]. The importance of pregnancy-related stress and coping strategies to counteract it contributes to psychiatric symptomatology during the current COVID-19 pandemic [22].

Maternal perception of social support may reduce prenatal stress and adverse psychological conditions during pregnancy, contributing to a reduced risk of postnatal affective symptoms [9,12,23]. In addition, pregnant women are concerned about the fetal growth and their future responsibilities and, therefore, are prone to various levels of psychological problems such as mood swings, fatigue, emotional disturbances, mixed anxiety disorder and depression, and pregnancy-related anxiety [6].

This research aims to describe women’s anxiety and stress during pregnancy in times of pandemic and its relationship with social support. For this purpose, we intend to relate these variables and compare whether there are differences between groups of pregnant women. These objectives will allow us to learn the behavior of a particular population and to promote aspects that may have an impact on the well-being of the pregnant woman and the fetus.

## 2. Materials and Methods

### 2.1. Study Design and Sample

A quantitative, descriptive, cross-sectional study was carried out using circumstantial non-probabilistic sampling. The study included pregnant women who attended the obstetrics office for the first time in their first trimester of gestation and who belonged to a Regional Management of the health system of Castilla y León (Spain). The exclusion criteria were: previous diagnosis of depression or anxiety, previously diagnosed psychiatric disease, language difficulties in the recruitment process, or lack of authorization or refusal to participate in the study. According to Castilla y León Health Services (SACYL) data, for the year 2020 (last year published), there were a total of 582 deliveries in the region under study [24].

A total of 150 first trimester pregnant women agreed to participate in the study, and the response rate to the questionnaires was 76.66% (*n* = 115). The mean age was 33.87 years, with a maximum of 47 years, a minimum of 20 years, and a mode of 35 years. A total of 52.2% (*n* = 60) of the women were multiparous while for 47.8% (*n* = 55), it was their first pregnancy. Nulliparous women with a previous first trimester abortion was considered primigravidae. Table 1 describes the sample according to the different socio-demographic variables analyzed.

All participants gave voluntary informed consent, which was carried out in accordance with the Declaration of Helsinki (World Medical Association, 2013) and the Good Clinical Practice Directive (Directive 2005/28/EC) of the European Union. The protocol was approved by the Ethics Committee of the University of León (ETICA-ULE-033-2021) and the Clinical Research Ethics Committee of the León and Bierzo Health Areas (reference code 21124).

### 2.2. Procedure

The sample was recruited in the first trimester of pregnancy at the obstetric control visit of the reference hospital of the health care management of SACYL. The data collection period was between September and December 2021. The Google Forms platform was used to prepare the survey. In a 5-min individual briefing session after the consultation with the obstetrician, the participants were invited to fill in the questionnaire by email prior to the informal consultation. The average time to complete the questionnaire was 10 min. Participants did not receive any type of incentive for their participation.

### 2.3. Instruments

The electronic form included a questionnaire with validated measurement instruments and sociodemographic data of the participants. The variables included were age, marital status, area of residence, previous obstetric history, and current pregnancy.

To measure anxiety, we used the Pregnancy Related Anxiety Questionnaire PRAQ-20 [25]. This scale measures five dimensions related to the anxiety that pregnant women feel about the fact that they are pregnant: concern for changes in oneself, fear for the integrity of the baby, feelings about oneself, fear of childbirth, and concerns about the future and their ability as a mother. Each item is scored from one to five points using a Likert scale (5 = strongly agree and 1 = strongly disagree). The reliability of the PRAQ-20 total scale was 0.91 in the first trimester, while the reliability values were 0.78 for concern for changes in oneself, 0.91 for fear for the baby’s integrity, 0.82 for feelings about oneself, 0.83 for fear of childbirth, and 0.71 for concern about the future, all for the first trimester of gestation. The reliability of this scale varies in nulliparous and multiparous women, being 0.92 in nulliparous women in the first trimester and 0.90 in multiparous women. The authors of the scale set the cut-off point at 67 points.

The social support variable was assessed through the Medical Outcomes Stud-Social Support Survey MOS-SSS [26]. This self-administered questionnaire evaluates the following items: social support network, social emotional/informational support, instrumental support, positive social interaction, and affective support. The Spanish validation of the MOS-SSS scale by the authors of the original scale showed a reliability of 0.94. Cronbach’s alpha values were 0.92 for emotional/informational social support, 0.79 for instrumental support, 0.83 for positive social interaction, and 0.74 for affective support.

Finally, stress was measured through the psychometric instrument Perceived Stress Scale (PSS) [27]; this instrument was designed to measure the degree to which situations in life are appraised as stressful. The Spanish version of the PSS (EER-14 items) demonstrated adequate reliability, validity, and sensitivity. Each item was scored from 0 to 4 points using a Likert scale (4 = very often and 0 = never). The reliability of this scale was 0.81.

### 2.4. Data Analysis

A descriptive analysis of the quantitative variables was performed by calculating the mean values and standard deviation. Qualitative variables were expressed as frequencies and percentages.

To analyze the associations between quantitative variables, the Pearson’s correlation coefficient was used. The relationship between quantitative and qualitative variables was determined by the Student’s *t*-test. To calculate the psychometric indicators of the measurement instruments used, the reliability coefficient (Cronbach’s alpha) was analyzed. Finally, multiple linear regression models were used for the perceived stress scale (PSS) as the dependent variable and the subscales of the anxiety (PRAQ-20) and social support (MOS-SSS) questionnaires as independent variables. Statistically significant results were established with a *p*-value < 0.05.

The SSPSS v.26 statistical package was used for data analysis.

## 3. Results

Table 2 shows the descriptive statistics of anxiety (PRAQ-20), social support (MOS-SSS), and stress (PSS) of the total sample and the mean differences with respect to the pregnancy status (multiparous or primiparous) of the women during their first trimester of pregnancy. The primigravidae (12.13 ± 4.94) showed higher levels of “fear of childbirth” than the multiparous group (8.92 ± 4.32). A statistically significant mean difference was also obtained for the PRAQ-20 dimension “concern about the future” between the primigravida group (4.80 ± 2.65) and the multiparous group (3.95 ± 1.53). Of the female participants, 78.3% presented anxiety with the PRAQ-20.

To examine the association between variables, Pearson’s correlation analysis was performed. Statistically significant correlations were found between stress and several variables, as shown in Table 3. Self-perceived stress correlates significantly with the dimensions “concern for changes in oneself”, “feelings about oneself”, and “concern about the future” of the PRAQ-20 and very weakly, with the total value of the MOS-SSS. In addition, a negative correlation was observed between the “feelings about oneself” of the PRAQ-20 and all dimensions of social support measured with the MOS-SSS.

To investigate the influence of the variables self-perceived stress and the dimensions of social support that correlate with “feeling about oneself” measured with the PRAQ-20, a linear regression analysis was performed. The results of the two models can be seen in Table 4. Model 2 explains 23.6% of the variance. Lower scores on self-perceived stress and higher scores on instrumental support predicted lower scores on feelings about oneself, which may result in lower scores on anxiety (PRAQ-20). In the case of parity, regression analysis showed three models represented in Table 4. Model 3 explained 19.2%. Multiparous women scored lower on fear of childbirth and emotional support and higher on fear for the integrity of baby than primiparous pregnant women.

## 4. Discussion

The aim of the present investigation was to describe women’s anxiety and stress during pregnancy in times of pandemic and its relationship with social support.

Regarding the questionnaires used, we observed that both the PRAQ-20 [25] and the MOS-SSS [26] showed high internal consistency, with Cronbach’s alpha values similar to those described in the literature. As for the PSS [27], we observed an important decrease in the Cronbach’s alpha value.

Pregnancy-related anxiety in our population was 78.3%, a high value in contrast to another similar study using the seventeen-item version of the PRAQ in which it was almost 21% of the participants [6]. The present study did not analyze data before the pandemic; however, scientific evidence points to an increase in the values of mental pathology in this population due to the epidemiological situation [4,5,6,12,28].

Within the socio-demographic variables and attending to parity, we observed that our population was relatively homogeneous in terms of multiparous and primiparous, 47.8% and 52.2%, respectively. The results of this research showed that the number of pregnancies is a factor to consider when analyzing anxiety related to pregnancy, unlike in other similar research, in which it is mentioned that having more children is a risk factor for having anxiety in pregnancy because these women have already experienced the worries of being pregnant [29,30]. The total values of pregnancy-related anxiety showed a non-statistically significant difference as a function of the participants’ parity. Some of the dimensions of the PRAQ-20 were altered and with statically significant differences, with “fear of childbirth” and “concern about the future” being the items that worried first-time pregnant women more than multiparous woman, in contrast to a similar study by Hamzehgardeshi et al. (2021), which showed that parity was a predictor of pregnancy-related anxiety, and that primiparous and multiparous women showed different values of anxiety [6]. Although the evidence repeatedly states that “fear of childbirth” is higher in primiparous women [7,8], the study by Dencker et al. (2019) showed that primiparous and multiparous women had similar levels of fear of childbirth [16]. The latter study relates its results to negative birth stories told by others, frightening information, or more mood-related problems in preterm women, while multitasking suggests that women report lower levels of fear if they have had an uncomplicated birth. Another explanation for the different results on parity may be due to the different definitions of fear of childbirth used [16]. In relation to women’s concern about fetal wellbeing and childbirth anxiety, the study by Taubman-Ben-Ari et al. (2021) [7] showed high values of concern. Pregnancy during pandemic is perceived as a risk in itself and for the integrity of their future babies [7,28]. Pregnancy-related anxiety and stress is a premise shared with several published studies [6,7,28]. The results of this study found that women with higher perceived stress were more likely to be anxious than those with lower perceived stress, which is consistent with a recent study [12].

As in other similar research on anxiety, stress is also related to parity, but inversely, since the greater the number of children, the lower the stress levels of women, however, in our study, we could not affirm this relationship, since in our population, primigravidae and multigestation women had similar levels of stress [29,30].

In addition, the participating pregnant women from SACYL management presented higher levels of self-perceived stress with higher values of social support. It is important to note that this population was exclusively made up of pregnant women in the first trimester and it is possible that they have not reflected on the importance of social support. This information contrasts with research by Grumi et al. (2021), Chasson et al. (2020), and Wang et al. (2021), who reported that the higher the perception of support, the lower the level of stress [3,9,23]. The literature reviewed on social support and stress showed populations of pregnant women in all three trimesters of pregnancy [3,9,23].

In the study by Awad-Sirhan et al. [29], an important variable in the appearance of stress and anxiety in pregnancy is related to the method by which the woman became pregnant, whether it was a spontaneous pregnancy or through assisted reproduction techniques; in our study this difference was not found, perhaps because almost 95% of our population had had a spontaneous pregnancy.

This study had some limitations that should be considered when interpreting the data. First, all enrolled subjects lived in a county in northern Spain and the results cannot be extended to other populations. Second, the study research was based solely on quantitative data collected through online methods using self-reporting questionnaires. Third, the designs related to the present study were correlational, and our results were cross-sectional. Therefore, longitudinal studies are recommended to investigate the causal relationship between the study variables. Finally, the small sample size could be a drawback when extrapolating the data. The various limitations of this study should lead to opportunities for future studies and it would be interesting to conduct studies in different trimesters of pregnancy in order to generalize the data and be able to be generalized to the general population of pregnant women.

## 5. Conclusions

During the COVID 19 pandemic, the anxiety levels of women in the SACYL regional management area were elevated. Higher levels of anxiety were related to higher self-perceived stress and vice versa. Increasing social support and decreasing stress seemed to influence the “feeling about oneself”, which may improve the anxiety values of this population. In addition, during the first trimester of pregnancy, primiparous women showed higher levels of fear of childbirth and emotional support and lower values of fear for the integrity of the baby than multiparous women.

Further research is needed in this population, where studies should be conducted in the remaining trimesters of pregnancy and health education resources should be developed to positively influence the well-being of the pregnant population throughout pregnancy.

## Figures and Tables

**Table 1:** Descriptive statistics of the sample.

Sociodemographic Variables		*n* = 115 (100%)
Parity	Primiparous	60 (52.2%)
	Multiparous	55 (47.8%)
Marital Status	Married/cohabiting	93 (80.9%)
	Single/widowed	22 (19.1%)
Abortions	None	92 (80%)
	One or more	23 (20%)
	None	77 (67%)
Childbirth	One	30 (26.1%)
	Two	5 (4.3%)
	Three or more	3 (2.6%)
Cesarean	None	101 (87.8%)
	One or more	14 (12.2%)
Area of residence	Urban	85 (73.9%)
	Rural	30 (26.1%)
Type of pregnancy	Spontaneous	109 (94.8%)
	Assisted reproduction	6 (5.2%)
Desired breastfeeding	Breastfeeding	56 (48.7%)
	Artificial lactation	13 (11.3%)
	Mixed breastfeeding	24 (20.9%)
	Unknown	22 (19.1%)

**Table 2:** Descriptive statistics of anxiety, social support, and stress of the simple and difference in means by gravity.

Questionnaires/Variables		Total Sample M ± ST	Multiparous M ± ST	Primiparous M ± ST	α	*t*	*p*
PRAQ-20	Concern for changes in oneself	7.11 ± 3.55	7.23 ± 3.87	6.98 ± 3.2	0.83	0.38	0.71
	Fear for the integrity of the baby	27.04 ± 7.82	27.20 ± 7.52	26.87 ± 8.21	0.91	0.22	0.82
	Feelings about oneself Fear of childbirth	6.67 ± 3.34	6.70 ± 3.28	6.64 ± 3.43	0.81	0.10	0.92
		10.45 ± 4.88	8.92 ± 4.32	12.13 ± 4.94	0.81	−3.72	0.00 *
	Concern about the future	4.36 ± 2.17	3.95 ± 1.53	4.80 ± 2.65	0.73	−2.12	0.04 *
	Total	55.63 ± 16.37	54 ± 14.74	57.42 ± 17.96	0.91	−1.12	0.27
MOS-SSS	Emotional/informational support	36.35 ± 6.38	35.42 ± 7.61	37.36 ± 4.53	0.96	−1.65	0.10
	Tangible support	17.90 ± 3.31	17.62 ± 3.66	18.22 ± 2.88	0.88	−0.97	0.10
	Positive social interaction	18.27 ± 3.46	18.00 ± 3.65	18.56 ± 3.25	0.91	−0.87	0.33
	Affective support	13.96 ± 2.32	13.87 ± 2.39	14.05 ± 2.25	0.86	−0.43	0.37
	Total	87.10 ± 13.56	85.33 ± 16.10	89.02 ± 9.89	0.97	−1.46	0.54
PSS	Total	32.68 ± 5.30	32.38 ± 4.43	33.00 ± 6.14	0.57	−0.62	0.51

Note: M ± ST: Mean ± Standard Deviation; t: *t*-test; α: Cronbach’s alpha; *: *p* < 0.05; PRAQ-20: Pregnancy Related Anxiety Questionnaire; MOS-SSS: Medical Outcomes Study-Social Support Survey; PSS: Perceived Stress Scale.

**Table 3:** Pearson inter-scale correlations between all variables included in the present study.

Questionnaires/Variables		PRAQ-20						MOS-SSS				
		CO	FB	FO	FC	CF	T	ES	TS	PS	AS	T
PRAQ-20	FB	0.354 **	1									
		0.000										
	FO	0.556 **	0.354 **	1								
		0.000	0.000									
	FC	0.318 **	0.584 **	0.432 **	1							
		0.000	0.000	0.000								
	CF	0.457 **	0.243 *	0.581 **	0.471 **	1						
		0.000	0.009	0.000	0.000							
	T	0.655 **	0.833 **	0.699 **	0.796 **	0.607 *	1					
		0.000	0.000	0.000	0.000	0.045						
MOS-SSS	ES	−0.128	0.164	−0.222 *	−0.042	−0.113	0.003	1				
		0.174	0.080	0.017	0.654	0.230	0.975					
	TS	−0.186 *	0.144	−0.245 *	0.004	−0.148	−0.040	0.863 **	1			
		0.046	0.125	0.008	0.963	0.115	0.671	0.000				
	PS	−0.074	0.109	−0.224 *	0.064	−0.078	−0.001	0.796 **	0.674 **	1		
		0.433	0.245	0.016	0.494	0.407	0.994	0.000	0.000			
	AS	−0.125	0.078	−0.266 **	−0.021	−0.122	−0.067	0.725 **	0.593 **	0.923 **	1	
		0.183	0.410	0.004	0.826	0.193	0.479	0.000	0.000	0.000		
	T	−0.153	0.155	−0.224 *	0.032	−0.127	−0.012	0.984 **	0.912 **	0.811 **	0.735 **	1
		0.102	0.098	0.016	0.734	0.175	0.896	0.000	0.000	0.000	0.000	
PSS	T	0.267 *	0.182	0.387 **	0.125	0.258 **	0.295 **	0.171	0.171	0.072	−0.019	0.185 *
		0.004	0.052	0.000	0.183	0.005	0.001	0.068	0.067	0.444	0.308	0.048

Note; *: *p* < 0.05: **; *p* < 0.01; CO: Concern for changes in oneself; FB: Fear for the integrity of the baby; FO: Feelings about oneself; FC: Fear of childbirth: CF: Concern about the future; T: Total; ES: Emotional/informational support; TS: Tangible support; PS: Positive social interaction; AS: Affective support.

**Table 4:** Lineal regression models.

Dependent Variable	Model	Variables Included	Beta	*p*	*t*	95% CI	Adjusted R^2^
Feelings about oneself	1	Perceived Stress Scale (PSS)	0.24 **	0.000	4.46	0.14–0.35	0.142
	2	Perceived Stress Scale (PSS)	0.28 **	0.000	5.31	0.18–0.38	0.236
		Tangible support (MOSS-SS)	−0.32 **	0.001	−3.86	−0.49–(−0.16)	
Parity	1	Fear of childbirth (FC)	0.03 **	0.000	3.72	0.02–0.05	0.101
	1	Fear of childbirth (FC)	0.05 **	0.000	4.93	0.03–0.08	0.164
	2	Fear for the integrity of the baby (FB)	−0.02 *	0.003	−3.08	−0.03–(−0.01)	
	1	Fear of childbirth (FC)	0.06 **	0.000	5.15	0.03–0.08	0.192
	2	Fear for the integrity of the baby (FB)	−0.02 **	0.001	−3.47	−0.04–(−0.01)	
	3	Emotional/informational support (ES)	0.02 *	0.028	2.23	0.00–0.03	

Note; Beta: regression coefficient; *: *p* < 0.05; **: *p* < 0.01; 95% CI: Confidence Interval; Adjusted R^2^: Adjusted coefficient of determination.

## Data Availability

The data processed in this article are part of a doctoral thesis in progress. They will be available on request from the corresponding author after exploitation.
